# The role of fat-free mass index in evaluating protein-energy wasting in maintenance hemodialysis patients: a multicenter cross-sectional study

**DOI:** 10.3389/fnut.2026.1743870

**Published:** 2026-05-08

**Authors:** Danhua Liang, Xianrui Dou, Yongqian Liang, Lan Liu, Wei Zhang, Ke Chen, Peiyi Li, Xu Lin, Rongshao Tan

**Affiliations:** 1 Department of Nutrition, The Eighth Affiliated Hospital, Southern Medical University (The First People's Hospital of Shunde, Foshan), Foshan, China; 2 Department of Nephrology, The Eighth Affiliated Hospital, Southern Medical University (The First People's Hospital of Shunde, Foshan), Foshan, China; 3 Department of Endocrinology and Metabolism, The Eighth Affiliated Hospital, Southern Medical University (The First People's Hospital of Shunde, Foshan), Foshan, China; 4 Guangzhou Institute of Disease-Oriented Nutritional Research, Guangzhou Red Cross Hospital of Jinan University, Guangzhou, China

**Keywords:** bioelectrical impedance analysis, fat-free mass index, hemodialysis, nutritional status assessment, protein-energy wasting

## Abstract

**Objective:**

Protein-energy wasting (PEW) is prevalent in maintenance hemodialysis (MHD) patients and associated with poor outcomes. This study aimed to evaluate the agreement of fat-free mass index (FFMI) measured by bioelectrical impedance analysis (BIA) with conventional nutritional tools and to explore its potential value as a supplementary screening measure for PEW in MHD patients.

**Methods:**

In this multicenter cross-sectional study, 272 MHD patients were enrolled between April 2016 and October 2019. Body composition was assessed using a multifrequency BIA device capable of segmental and whole-body composition analysis. Patients were classified into low or normal FFMI groups based on established sex-specific criteria (FFMI < 17 kg/m^2^ for men and < 15 kg/m^2^ for women). Nutritional status was further evaluated using serum albumin (ALB), body mass index (BMI), Nutritional Risk Screening 2002 (NRS2002), and the Seven-Point Subjective Global Assessment (7-point SGA). Correlation and agreement analyses were performed between FFMI and other nutritional parameters.

**Results:**

The mean age of participants was 58.8 ± 12.7 years, with a mean FFMI of 16.6 ± 2.2 kg/m^2^. The prevalence of low FFMI was 38.6%. FFMI demonstrated a non-linear relationship with age, peaking in the 41–50 age group. It was positively correlated with body weight (*r* = 0.71, *p* < 0.01), BMI (*r* = 0.59, *p* < 0.01), mid-arm circumference (MAC, *r* = 0.55, *p* < 0.01), mid-arm muscle circumference (MAMC, *r* = 0.52, *p* < 0.01), and handgrip strength (HGS, *r* = 0.24, *p* < 0.01). Notably, FFMI identified a significant proportion of patients with low muscle mass who were classified as normal by ALB (38.7%), BMI (31.4%), NRS2002 (23.4%), and 7-point SGA (20.0%). The McNemar test and consistency analysis revealed fair to moderate agreement between FFMI and BMI, NRS 2002, and the 7-point SGA (*p* < 0.01).

**Conclusion:**

Conventional nutritional assessment tools, including ALB, BMI, NRS2002, and SGA, frequently fail to detect low muscle mass in MHD patients. FFMI derived from BIA provides an objective, non-invasive, and potentially useful measure that may aid in the screening of PEW, particularly in detecting low muscle mass that is often missed by conventional tools. We recommend the integration of BIA-based body composition analysis as a supplementary tool into routine nutritional management of hemodialysis patients to enable timely interventions.

## 1 Introduction

Protein-energy wasting (PEW), characterized by the loss of lean body mass and fat reserves, is a condition of malnutrition specific to dialysis patients (1). PEW is commonly observed in maintenance hemodialysis (MHD) patients, with prevalence rates ranging from 18 to 75%, depending on the methods used to assess nutritional status (2, 3). Recent study has demonstrated that PEW contributes to sarcopenia in MHD patients by reducing muscle mass and strength (4), which is associated with a diminished quality of life and an elevated risk of mortality (5, 6). According to the Kidney Disease Outcomes Quality Initiative (KDOQI), the diagnosis of PEW requires meeting at least 3 of 4 core criteria: low serum protein markers, reduced anthropometric measures, inadequate dietary intake, and evidence of muscle mass loss (7). Therefore, the accurate screening of PEW with low muscle mass and timely implementation of nutritional intervention are crucial in MHD patient management.

Current guidelines recommend Nutritional Risk Screening 2002 (NRS2002) as a nutritional risk screening tool and Seven-point Subjective Global Assessment (7-point SGA) as a nutritional assessment tool in hemodialysis patients (7–9). However, these tools rely on subjective clinician judgment, potentially compromising their reproducibility and objectivity. Furthermore, NRS2002 does not include muscle mass assessment. While body mass index (BMI) and albumin (ALB) are commonly used as objective nutritional assessment indicators (7), they are also unable to identify muscle loss. Additionally, BMI is easily affected by body water levels in the state of fluid overload, while albumin also has limitations as a nutritional marker due to its susceptibility to inflammation. Therefore, there is a pressing need for a simple, objective, and potentially useful method that includes objective assessment of muscle mass to facilitate the identification of PEW in clinical practice, particularly in Chinese dialysis centers, where the hemodialysis population is large and resources can vary, highlighting the need for practical and cost-effective tools.

Bioelectrical impedance analysis (BIA) is a technology that leverages the electrical characteristics and changes of biological tissues and organs to extract biomedical information about human body composition (10), and it has proven to be a simple, quick, objective, and noninvasive method. It assumes that the human body is composed of fat mass (FM) and fat-free mass (FFM). BIA measures impedance, resistance, and reactance of an electric current traveling through the body and estimates total body water (TBW), fat-free mass (FFM) and fat mass (FM) (11, 12). The fat-free mass index (FFMI), calculated as FFM divided by height squared (kg/m^2^), standardizes FFM for stature and is a surrogate indicator of skeletal muscle mass. Guidelines from the European Society for Clinical Nutrition and Metabolism (ESPEN) and the Global Leadership Initiative on Malnutrition (GLIM) endorse FFMI as a core criterion for diagnosing malnutrition (13, 14).

However, the practical utility and screening value of the FFMI in MHD patients remains to be fully illustrated. Hence, this study aimed to investigate the agreement between FFMI (measured by BIA) and conventional nutritional tools, and to explore its potential value as a supplementary screening measure for PEW in maintenance hemodialysis patients.

## 2 Materials and methods

### 2.1 Study design and participants

This cross-sectional study was conducted at the Blood Purification Center of three tertiary hospitals between April 2016 and October 2019. This was a cross-sectional study with a single assessment time point for all measurements. The study was approved by the Ethics Committee of the hospital (approval number 2014-057-01), and informed consent was obtained from all participating patients.

*A priori* sample size calculation was conducted for this cross-sectional study. Based on a previously reported prevalence of protein-energy wasting (PEW) of 34.1% in a similar Chinese maintenance hemodialysis population (15), a sample size estimation was performed using the formula for estimating a single population proportion: *n* = (*Z*^2^ × *p* × (1 − *p*)/*d*^2^), where *Z* is the *Z*-value for the desired confidence level (1.96 for 95% CI), *p* is the expected prevalence (0.341), and *d* is the margin of error (precision). Setting the margin of error (*d*) at 0.06 (6%), the calculated minimum required sample size was 241. Anticipating a potential non-response or data incompleteness rate of approximately 10–15%, the target sample size was adjusted to approximately 270–280. Consequently, our final enrolled sample of 272 patients meets and exceeds the minimum statistical requirement, ensuring sufficient precision for estimating the prevalence of low FFMI and associated nutritional parameters in the study population.

Inclusion criteria were: (1) aged 18–80 years; (2) receiving regular hemodialysis (three times per week, 4 h each session) for more than 3 months before enrolment. The exclusion criteria included: (1) serious infections within the past 3 months; (2) recent surgical treatment that might influence nutritional status; (3) individuals with contraindications for BIA, such as limb amputation or the presence of a pacemaker; (4) acute poor appetite, eating disorder, or hospitalization within 1 month before enrolment that could acutely affect nutritional status.

### 2.2 Bioelectrical impedance analysis measurement

Body composition was assessed using the InBody S10 (Biospace, Seoul, Korea), a multifrequency BIA device capable of segmental and whole-body composition analysis. To estimate fat-free mass (FFM) and other parameters, the device applies a small alternating electrical current at six specific frequencies (1, 5, 50, 250, 500, and 1,000 kHz) through an eight-point tactile electrode system. The proprietary, manufacturer-programmed multi-frequency bioelectrical impedance spectroscopy equations of the InBody S10 were used to derive body composition estimates. The BIA measurement of dialysis patients was conducted by the same operator 30 min after the completion of a hemodialysis session, a time interval generally accepted to allow for stabilization of extracellular fluid distribution and reduction of intradialytic hemodynamic fluctuations (7, 16), following a standardized resting period. BIA-derived fat mass (FM), fat-free mass (FFM), total body water (TBW), and extracellular water (ECW) values were recorded. FFMI was calculated by the following equation: FFMI = Fat-free mass (kg)/Height^2^ (m^2^). Patients were stratified into low FFMI and normal FFMI groups based on ESPEN criteria: FFMI < 17 kg/m^2^ for men and <15 kg/m^2^ for women (13). To address potential confounding from hydration status, the ultrafiltration volume for the dialysis session preceding the BIA measurement was recorded, and the TBW-to-FFM percentage and ECW-to-TBW ratio were calculated as objective hydration indicators.

### 2.3 Laboratory measurements

The blood samples were collected from each patient with fasting before the HD session and sent for biochemical examination in the hospital laboratory. Serum levels of creatinine (Cr), total cholesterol (TC), triglycerides (TG), prealbumin (PA), albumin (ALB), and hypersensitive C-reactive protein (hs-CRP) were measured using a Hitachi 7600 automatic biochemical analyzer (Hitachi, Tokyo, Japan). The efficiency of dialysis was assessed based on the delivered dose of dialysis Kt/V using the natural logarithm formula of Daugirdas, where *K* is the dialysis clearance measured by the pre- and post-dialysis urea levels, *t* is the duration of dialysis in minutes, and *V* is the volume of distribution of urea, estimated as 60% of body weight.

### 2.4 Anthropometric measurements

Anthropometric measurements and BIA measurement were performed on the same day. The MHD patients were measured for height and dry weight (post-dialysis weight) by the electronic column scales (SECA 206, Seca, Germany). Body mass index (BMI) was calculated as weight (kg) divided by height squared (m^2^). Handgrip strength (HGS) was measured on the non-fistula arm using a Jamar hydraulic hand dynamometer (Sammons Preston, Masan, Korea), and the best of three attempts was recorded. Mid-arm circumference (MAC) was measured with a flexible, non-stretchable measuring tape while the triceps skinfold (TSF) was measured by the Harpenden skinfold caliper (Baty International, West Sussex, UK) on the non-fistula arm for hemodialysis patients. MAC and TSF measurements were performed in triplicate by a single trained assessor, and the average value was used for analysis. Mid-arm muscle circumference (MAMC) was calculated from these measurements using the formula: MAMC (cm) = MAC (cm) − *π* × TSF (cm).

### 2.5 Identification of protein-energy wasting

BMI < 18.5 kg/m^2^ indicates malnutrition (BMI+). Albumin < 35 g/L is considered low albumin (Albumin+). Nutritional risk is screened using the NRS 2002, with a score ≥ 3 indicating nutritional risk (NRS 2002+). The 7-point SGA is utilized for nutritional assessment, and 7-point SGA score ≤ 5 is diagnosed as PEW (7-point SGA+) (9). According to the cut-off values established by ESPEN (13), FFMI of <17 kg/m^2^ for men or <15 kg/m^2^ for women is defined as low FFMI (FFMI+).

### 2.6 Dietary intake

Food and beverage consumption was assessed from 24-h dietary recall questionnaires (17) through face-to-face interviews for 3 days, comprising two non-consecutive dialysis days and one non-dialysis day. The energy intake of all food and drink items was sourced using a computer-aided dietary software (Zhending, Shanghai, China; software version 2.0), in which nutrient models were according to the Chinese Food Composition Table came from the Chinese Center for Disease Control and Prevention in 2018. Based on Kidney Disease Outcomes Quality Initiative (KDOQI) guidelines, inadequate intake was defined as energy intake <30 kcal/kg/day or protein intake <1.0 g/kg/day (7).

### 2.7 Statistical analysis

Statistical analyses were performed using SPSS version 21.0 (IBM Corp., Armonk, NY, USA). The normality of continuous variables was assessed using histograms and Shapiro–Wilk tests. Normally distributed data are presented as mean ± standard deviation (SD) and compared using independent samples t-tests. Non-normally distributed data are presented as median (interquartile range, IQR) and compared using the Mann–Whitney U test. Categorical variables are expressed as frequencies (percentages) and compared using the chi-square test. Correlations between FFMI and continuous nutritional variables were examined using Pearson’s correlation coefficient. The agreement between FFMI (dichotomized) and other nutritional assessment tools (BMI, ALB, NRS2002, 7-point SGA) was evaluated using the McNemar test and Cohen’s kappa statistic. Agreement was interpreted as: <0 = no agreement, 0.01–0.20 = slight agreement, 0.21–0.40 = fair agreement, 0.41–0.60 = moderate agreement, 0.61–0.80 = substantial agreement, 0.81–1.00 = almost perfect agreement (18). A two-sided *p* value < 0.05 was considered statistically significant.

## 3 Results

### 3.1 General characteristics

A total of 272 MHD patients were included in the final analysis. The mean age was 58.8 ± 12.7 years, and 53.7% (*n* = 146) were male. The majority of the HD patients (79.7%) had been on dialysis for <5 years. The most common primary causes of end-stage renal disease were diabetes mellitus (27.2%), hypertension (18.0%) and chronic glomerulonephritis (17.6%). The mean Kt/V was 1.29 ± 0.25, indicating adequate dialysis. The hydration status at the time of BIA measurement was also assessed. The mean ultrafiltrate volume was 2.71 ± 1.10 L. The mean TBW/FFM percentage was (73.62 ± 0.39)%, and the mean ECW/TBW ratio was 0.39 ± 0.02. The mean FFMI was 16.6 ± 2.2 kg/m^2^. Notably, 45.2% of patients reported inadequate dietary intake. The baseline characteristics of the study participants are summarized in Table 1.

**Table 1:** Baseline characteristics of hemodialysis patients (*n* = 272).

Characteristics	*n* (%)*
Age (years, mean ± SD)	58.79 ± 12.73
Male (%)	146 (53.7%)
Primary cause of kidney failure (%)	
Chronic glomerulonephritis	48 (17.6%)
Diabetes mellitus	74 (27.2%)
Hypertension	49 (18.0%)
Unknown	30 (11.2%)
Others	71 (26.0%)
Dialysis duration (years) (%)	
<5 years	217 (79.7%)
≥5 years	55 (20.3%)
Dialysis adequacy (Kt/V, mean ± SD)	1.29 ± 0.25
Food intake	
Sufficient	149 (54.8%)
Insufficient	123 (45.2%)
FFMI (kg/m^2^, mean ± SD)	16.61 ± 2.17
Ultrafiltrate volume (L, mean ± SD)	2.71 ± 1.10
TBW/FFM percentage (%, mean ± SD)	73.62 ± 0.39
ECW/TBW ratio (mean ± SD)	0.39 ± 0.02

FFMI, fat-free mass index; TBW, total body water; FFM, fat-free mass; ECW, extracellular water.

*Unless specified.

### 3.2 Baseline characteristics and nutritional status by FFMI stratification

In the analysis of baseline characteristics and nutritional indicators stratified by FFMI, we found that older age and weight loss were more prevalent in the low FFMI group, with statistically significant differences (*p* < 0.05). In terms of nutritional status, there was a statistically significant increase in MAC, MAMC, and HGS in the FFMI normal group (*p* < 0.05), while there was no statistically significant difference in ALB, PA, TC, TG, hs-CRP, Ultrafiltrate volume, TBW/FFM percentage and ECW/TBW between the two groups (Table 2).

**Table 2:** Comparison of characteristics and nutritional status between two groups among the hemodialysis patients (*n* = 272).

Parameters	Low FFMI group (*n* = 105)	Normal FFMI group (*n* = 167)	*t/χ^2^/Z*	*p*
Age (years)	60.84 ± 14.25	57.50 ± 11.53	−2.12	0.04
Gender (*n*, %)				
Male	57 (61.00%)	89 (39.00%)	0.03	0.87
Female	48 (61.9%)	78 (38.1%)		
% of weight loss (*n*, %)				
<10%	90 (36.0%)	162 (64.0%)	39.1	<0.01
≥10%	15 (75.0%)	5 (25.0%)		
Dialysis duration (months)	30.50 (13.00–63.50)	25.00 (11.00–41.00)	−1.73	0.08
ALB (g/L)	38.39 ± 4.83	38.12 ± 5.43	−0.42	0.68
PA (mg/L)	333.95 ± 89.24	349.82 ± 59.61	1.30	0.19
TG (mmol/L)	1.23 (0.89–1.72)	1.20 (0.86–1.70)	−3.59	0.72
TC (mmol/L)	4.23 ± 1.16	4.23 ± 1.14	0.50	0.62
hs-CRP (mg/L)	2.43 (1.10–5.00)	3.10 (1.20–5.00)	0.47	0.60
BMI (kg/m^2^)	19.67 ± 2.31	22.60 ± 3.12	−2.34	0.82
MAC (cm)	25.35 ± 2.64	26.30 ± 3.11	8.08	<0.01
MAMC (cm)	19.64 ± 2.88	22.25 ± 3.19	6.79	<0.01
HGS (kg)	14.30 (9.65–19.50)	19.20 (14.55–26.40)	2.57	<0.01
Ultrafiltrate volume (L)	2.51 ± 1.17	2.82 ± 1.05	−1.29	0.20
TBW/FFM (%)	73.58 ± 0.37	73.64 ± 0.40	−1.17	0.24
ECW/TBW	0.397 ± 0.013	0.394 ± 0.017	1.31	0.19

ALB, albumin; PA, prealbumin; TG, triglycerides; TC, total cholesterol; hs-CRP, hypersensitive C-reactive protein; BMI, body mass index; MAC, mid-arm circumference; MAMC, mid-arm muscle circumference; HGS, handgrip strength; TBW, total body water; FFM, fat-free mass; ECW, extracellular water.

### 3.3 Correlations between FFMI and age

**Figure 1:**
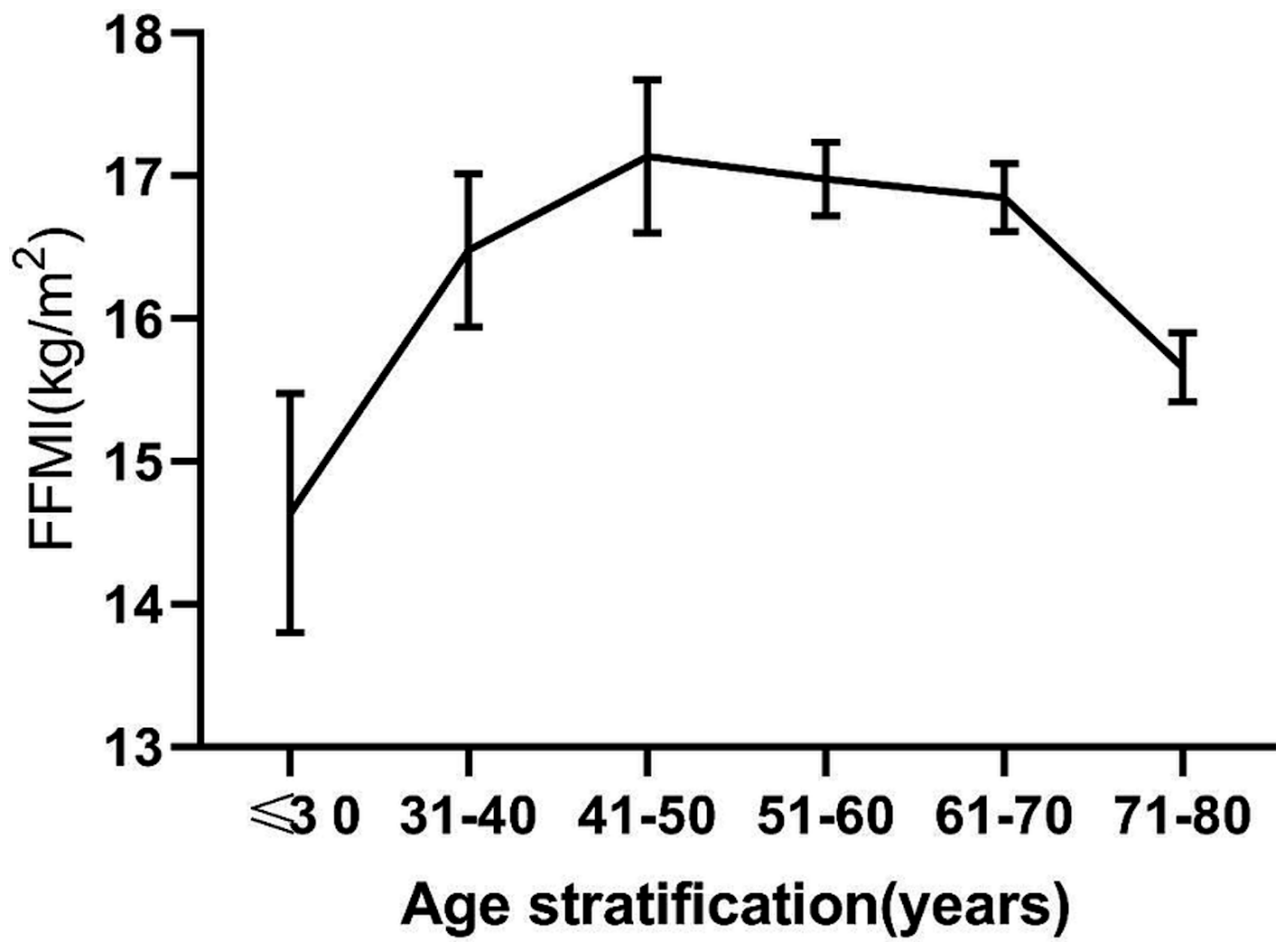
Changes of FFMI value stratified by age.

**Figure 2:**
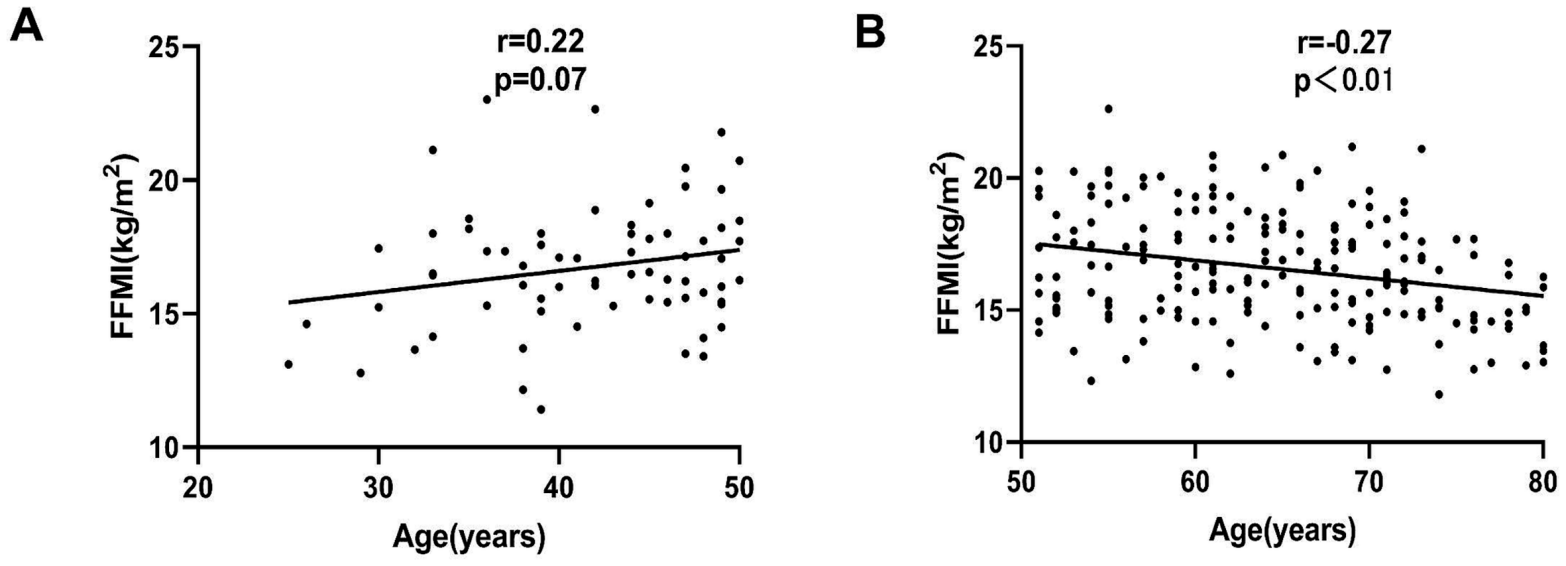
Regression linear relationship between age and FFMI in **(A)** patients aged 18–50 y and **(B)** patients aged 51–80 y.

We found that FFMI followed an inverse U-curve with age, rising to a peak in the 41–50 age group and decreasing afterward. FFMI was lower in patients aged <30 years or >70 years (Figure 1). According to the trend of the curve in Figure 1, we separated subjects into two age groups as follows: 18–50 y and 51–80 y. Further Pearson correlation analysis showed that the FFMI significantly decreased with age in patients aged 51–80 years (*r* = −0.27, *p* < 0.01). However, there was no correlation between FFMI and age in the 18–59 y group (*r* = 0.22, *p* = 0.07) (Figure 2).

### 3.4 Correlations between FFMI and nutritional indicators

Analysis of the correlation between FFMI and nutritional indicators in hemodialysis patients showed that FFMI was positively correlated with body weight (*r* = 0.71, *p* < 0.01), BMI (*r* = 0.59, *p* < 0.01), MAC (*r* = 0.55, *p* < 0.01), MAMC (*r* = 0.52, *p* < 0.01), HGS (*r* = 0.24, *p* < 0.01). However, no significant association was found between the FFMI with ALB, PA, FM, and TC (*p* > 0.05) (Table 3).

**Table 3:** Correlations between FFMI with nutritional indicators and hydration status Indicators (*n* = 272).

Parameters	*r*	*p*
ALB (g/L)	−0.01	0.85
PA (mg/L)	0.08	0.20
TC (mmol/L)	−0.02	0.78
Body weight (kg)	0.71	<0.01
BMI (kg/m^2^)	0.59	<0.01
FM (kg)	0.02	0.85
MAC (cm)	0.55	<0.01
MAMC (cm)	0.52	<0.01
HGS (kg)	0.24	<0.01
Ultrafiltrate volume (L)	0.36	<0.01
TBW/FFM (%)	0.19	<0.01
ECW/TBW	−0.21	0.01

ALB, albumin; PA, prealbumin; TC, total cholesterol; BMI, body mass index; FM, fat mass; MAC, Mid-arm circumference; MAMC, mid-arm muscle circumference, HGS, handgrip strength; TBW, total body water; FFM, fat-free mass; ECW, extracellular water.

### 3.5 Correlations between FFMI and hydration status indicators

The correlation between these hydration indicators and FFMI was analyzed (Table 3). A weak positive correlation was found between TBW/FFM and FFMI (*r* = 0.19, *p* < 0.01), which is physiologically expected as TBW is a major component of FFM. FFMI also showed a moderate positive correlation with ultrafiltrate volume (*r* = 0.36, *p* < 0.01), suggesting that patients with higher lean mass tended to have a greater amount of fluid removed during dialysis, potentially reflecting their larger baseline fluid volume capacity. In contrast, a weak negative correlation was observed between ECW/TBW and FFMI (*r* = −0.21, *p* = 0.01).

### 3.6 Consistency between FFMI and nutritional assessment scales/indicators

The prevalence of low FFMI (38.6%) was comparable to the prevalence of nutritional risk identified by NRS2002 (41.9%) and malnutrition diagnosed by 7-point SGA (38.6%), but was substantially higher than the prevalence of low BMI (16.9%) and hypoalbuminemia (4.0%) (Table 4).

**Table 4:** Consistency of the fat-free mass index (FFMI) with the nutritional scales/indicators in the nutritional assessment (*n* = 272).

BIA		Total	FFMI		Kappa	*p*
			+	−		
BMI	+	46 (16.9)	34 (73.9)	12 (26.1)	0.28	<0.01
	−	226 (83.1)	71 (31.4)	155 (68.6)		
Albumin	+	11 (4.0)	4 (36.4)	7 (63.6)	−0.01	0.88
	−	261 (96.0)	101 (38.7)	160 (61.3)		
NRS2002	+	114 (41.9)	68 (59.6)	46 (40.4)	0.37	<0.01
	−	158 (58.1)	37 (23.4)	121 (76.6)		
7-point SGA	+	105 (38.6)	72 (67.3)	35 (32.7)	0.47	<0.01
	−	167 (61.4)	33 (20.0)	132 (80.0)		
Total			105 (38.6)	167 (61.4)		

**Figure 3:**
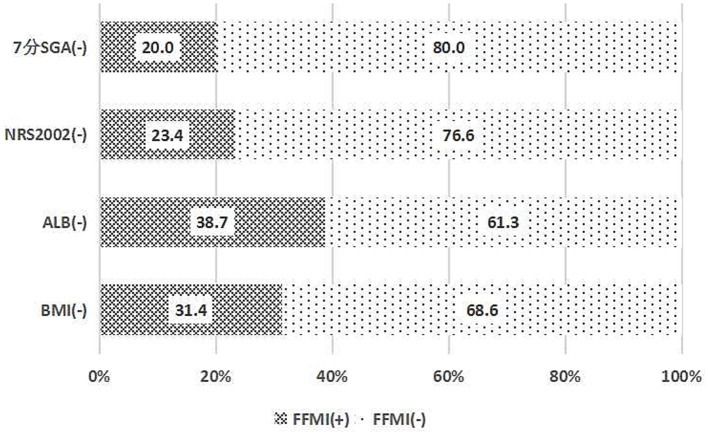
Consistency of the fat-free mass index with the nutritional scales/parameters in the nutritional assessment of hemodialysis patients.

Among patients with a BMI ≥ 18.5 kg/m^2^, 31.4% exhibited low FFMI. The rate of low FFMI was even higher at 38.7% among patients with albumin levels ≥35 g/L. Among those with a nutritional risk screening score of ≤3 according to the NRS 2002, the prevalence of low FFMI was 23.4%. Additionally, among patients without malnutrition as assessed by the 7-point SGA, the rate of low FFMI was 20.0% (Figure 3).

Consistency analysis revealed statistically significant but fair to moderate agreement between FFMI and BMI (Kappa = 0.28, *p* < 0.01), NRS2002 (Kappa = 0.37, *p* < 0.01), and 7-point SGA (Kappa = 0.47, *p* < 0.01). There was no significant agreement between FFMI and ALB (Kappa = −0.01, *p* = 0.88) (Table 4).

## 4 Discussion

The incidence of complications, infection, hospitalization, and adverse prognosis in CKD patients with PEW, especially in MHD patients, has significantly increased. PEW has become an independent risk factor affecting the survival of MHD patients (19–21). Therefore, the accurate screening of PEW in MHD patients is of great significance for improving prognosis. As per the KDOQI guidelines, the diagnosis of PEW requires meeting at least 3 out of 4 core criteria (low serum protein markers, reduced anthropometric measures, inadequate dietary intake, and evidence of muscle mass loss) (9). Hence, effective screening tools are needed to reliably assess these individual criteria, particularly muscle mass. It is critical to emphasize that FFMI is not intended as a standalone diagnostic tool for PEW, but rather serves as a highly effective complementary anthropometric indicator. It may assist in identifying the anthropometric criterion within a multidimensional assessment, without implying that it, by itself, validates or establishes the diagnosis of PEW. Unlike other anthropometric measures (e.g., BMI) that fail to distinguish muscle mass from fat mass or fluid status, FFMI specifically quantifies fat-free mass (the primary component of muscle mass), making it a more targeted indicator for supporting the screening of PEW. Its added value lies in being used in conjunction with subjective (NRS2002, 7-point SGA) and objective (ALB, dietary intake) tools: when combined with evidence of inadequate intake and low serum proteins, FFMI can support the screening of PEW in a multidimensional framework. This integrated approach addresses a critical clinical gap in current nutritional evaluation workflows, ultimately leading to a more comprehensive, objective, and rigorous screening process for PEW.

The present study recommends BIA as a valuable supplementary tool for the screening of PEW among MHD patients because of its advantages of being noninvasive, relatively simple, and inexpensive (7, 22). Studies suggested that low lean body mass and sarcopenia are common in MHD patients with PEW (4, 22). In recent years, BIA-derived FFMI, as an index of fat-free mass and a surrogate indicator of skeletal muscle mass, has been increasingly used as a supplementary measure within nutritional screening frameworks in clinical research. According to the PEW proposed by the ISRNM in 2008, the decline of muscle mass is one of the four diagnostic criteria. ESPEN suggested that the loss of muscle mass is a selective criterion for diagnosing malnutrition and defined a low FFMI as below 15 or 17 kg/m^2^ for females or males, respectively, in 2015 (13). The Global Leadership Initiative on Malnutrition (GLIM) includes low FFMI as a phenotypic criterion within its diagnostic framework for malnutrition, in combination with other etiologic criteria (14). However, it is important to note that while international guidelines recognize the clinical relevance of FFMI, the present study focuses on its utility as a supplementary screening tool in the specific context of MHD patients, rather than establishing a diagnostic protocol. More and more studies have shown that decreased muscle mass and function in maintenance hemodialysis patients are associated with poor prognosis (23–25). Currently, the commonly used nutritional screening parameters in China, such as BMI, albumin, NRS2002, and 7-point SGA do not provide an objective assessment of muscle mass. Therefore, BIA-derived FFMI has the potential to be a supplementary screening measure for the screening of PEW in hemodialysis patients, used in conjunction with other conventional tools.

In the study, it was found that the FFMI normal group had a lower age and fewer patients with weight loss of ≥10% when compared with the low FFMI group, indicating that an increase in age and weight loss in hemodialysis patients increases the risk of further muscle mass loss. A further analysis found that FFMI is a growing polarization of trends with age, rising to a peak in the 41–50 age category and decreasing afterward. In addition, FFMI decreased significantly with age in 51–80 y group because of the loss in the proportion of body water and the decline of muscle mass with the increase of age and the aggravation of the disease, which is consistent with other studies (26, 27). In terms of nutritional status, the body weight, MAC, MAMC, and HGS in the normal FFMI group significantly increased, which further supports the convergent validity of FFMI as a complementary anthropometric indicator. Luo (28) found that FFMI is closely related to the nutritional status and muscle function of patients with chronic obstructive pulmonary disease. The correlation analysis of this study also showed that FFMI is related to muscle mass indicators (MAC, MAMC, and HGS), indicating that FFMI can reflect the nutritional status and is more closely related to the lean tissue mass in dialysis patients. Studies already revealed that muscle wasting was commonly observed in MHD patients with PEW due to conditions such as inflammation, acidosis, and catabolic conditions (7, 22, 29), but it is important to note that the cross-sectional design of this study does not allow us to establish a causal relationship between muscle mass loss and PEW—we can only confirm a significant association between low FFMI and PEW-related phenotypic changes (e.g., weight loss, poor nutritional status) at the same time point. PEW and sarcopenia share overlapping phenotypic features, including muscle mass loss, and their co-occurrence in MHD patients likely reflects common underlying pathways such as inadequate protein intake and chronic inflammation; however, the temporal and causal direction of these relationships cannot be established from the present cross-sectional data.

A critical methodological consideration in BIA-based body composition assessment is hydration status, as fluid overload or depletion can alter electrical impedance and bias FFM estimation—particularly relevant in MHD patients who are prone to chronic fluid variability (11, 25). Our study addressed this concern by collecting multiple objective hydration indicators (TBW/FFM percentage, ultrafiltrate volume, ECW/TBW ratio) and providing indirect evidence that the study sample was likely in a relatively stable hydration state at the time of measurement. The mean TBW/FFM percentage (73.62 ± 0.39)% was consistent with euhydration in MHD patients, 95% of patients had ultrafiltrate volume within 1.0–4.0 L (no extreme fluid overload or depletion), and the mean ECW/TBW ratio (0.39 ± 0.02) fell within the normal range for stable hemodialysis patients. Furthermore, no significant differences in these hydration indicators were observed between the low FFMI and normal FFMI groups, reducing the likelihood that hydration variability acted as a major confounding factor in the comparison of FFMI. This is a key strength of our study, as it suggests that the observed differences in FFMI between groups reflect true differences in muscle mass rather than hydration-related measurement bias. It should be noted, however, that the proprietary equations used by the InBody S10 have not been independently validated in Chinese maintenance hemodialysis patients. Although widely used in similar populations, the lack of population-specific validation introduces potential bias and uncertainty in FFM estimation. Since FFMI is directly derived from FFM, this limitation may affect not only absolute values but also classification outcomes, and should therefore be carefully considered when interpreting FFMI-based comparisons.

Correlation analyses further quantified the relationship between FFMI and nutritional/hydration status. FFMI was moderately positively correlated with BMI (*r* = 0.59, *p* < 0.01), which is partially attributable to mathematical structural correlation: both indices share height^2^ as the denominator, and FFM is a direct component of body weight (the numerator of BMI). Thus, part of the observed correlation does not solely reflect a physiological association between FFMI and BMI, and this limitation should be recognized to avoid overinterpretation of the results. Notably, despite this structural overlap, FFMI still provides unique value because it specifically isolates fat-free mass, whereas BMI conflates fat mass, muscle mass, and fluid status—explaining why FFMI identified 31.4% of patients with normal BMI as having low muscle mass. FFMI was weakly positively correlated with TBW/FFM percentage (*r* = 0.19, *p* < 0.01), which is attributable to the fact that FFM itself contains a large proportion of water (an inherent physiological characteristic of lean tissue) rather than hydration disturbance. The moderate positive correlation between FFMI and ultrafiltrate volume (*r* = 0.36, *p* < 0.01) likely reflects the larger fluid retention capacity of patients with greater lean mass—since muscle tissue has a high water content, patients with higher FFMI may have a larger baseline fluid volume, leading to greater ultrafiltrate volume during dialysis. Additionally, FFMI was weakly negatively correlated with ECW/TBW ratio (*r* = −0.21, *p* = 0.01), suggesting that patients with higher muscle mass have better extracellular fluid distribution—consistent with the physiological role of skeletal muscle in maintaining fluid balance by sequestering water within cells. Collectively, these findings support that FFMI is a surrogate indicator of muscle mass in MHD patients, even in the context of typical hydration variability observed in clinical practice.

Previous studies have shown ALB is a generally used nutritional marker in CKD patients and is strongly associated with patient outcomes (30). However, our study found that 38.7% of patients with ALB ≥ 35 g/L—the threshold defined in our Methods—presented low FFMI, reinforcing that albumin levels within the normal range do not preclude the presence of muscle depletion. It may be that ALB is a marker of visceral protein, susceptible to be affected by fluid status and inflammation (31). Additionally, ALB takes several weeks before being reduced, rendering it a late indicator of malnutrition. Recent study also found that albumin concentrations measured in patients at hospital admission to be helpful to predict clinical outcomes among patients at nutritional risk, but it was not beneficial in predicting treatment response to nutritional intervention (32). Therefore, ALB poorly responds to changes in protein and muscle mass timely.

In addition, ESPEN guidelines in 2008 recommended that patients with a BMI ≤ 18.5 kg/m^2^ and poor general condition be evaluated for malnutrition. Many people in China will still use this standard to evaluate malnutrition. Our study showed that 31.4% of patients with a normal BMI have a lower FFMI. Further consistency analysis revealed a poor agreement between FFMI and BMI, which is partially influenced by the structural correlation between the two indicators as discussed earlier. This discrepancy arises because BMI fails to distinguish between an excess of body fat (BF) and an increase in fat-free mass (FFM), potentially leading to nutritional misclassification (33). Consequently, patients with similar BMI values can exhibit distinct body compositions and markedly different metabolic profiles. A 6-year observational study found that mortality was higher in hemodialysis patients with 20 < BMI < 25 than in patients with BMI < 20 when they had muscle weakness (33). Other studies have also shown that normal weight or even obesity hemodialysis patients with decreased muscle mass may predict higher morbidity and a high risk of falls (34). Therefore, BMI is not a tool for individual assessment but rather a population-level indicator.

Meanwhile, 23.4% of patients screened for no nutritional risk by NRS2002 had a lower FFMI. It means that if we only use NRS2002, we may ignore some patients with lower muscle mass. Compared with NRS2002, the 7-point SGA scale can detect earlier and more easily monitor the small improvements or deteriorations within each category and was considered to be a tool for PEW diagnosis (9). Our study found that 20% of patients with normal nutritional status assessed by a 7-point SGA had low FFMI, and further consistency analysis indicated that FFMI had moderate levels of concordance with a 7-point SGA. The moderate agreement observed between FFMI and the 7-point SGA is partly explained by conceptual overlap, as both instruments assess muscle mass—one objectively and the other through subjective clinical observation. However, this concordance should not be interpreted as evidence of independent validity or clinical superiority of FFMI over SGA. In the absence of an external reference standard such as DXA, the degree to which each instrument correctly classifies patients remains uncertain. The observed agreement reflects convergence on the same biological construct rather than independent corroboration of screening accuracy. The proportion of patients identified exclusively by FFMI (20% of those with normal 7-point SGA) may indicate differences in sensitivity between methods; however, in the absence of an independent reference standard, it is not possible to determine whether these cases represent true positives. Nonetheless, FFMI provides an objective, quantitative measure of muscle mass that can supplement the subjective clinical judgment inherent in SGA. Therefore, this study suggests that FFMI could serve as a complementary indicator for screening PEW in dialysis patients, used in conjunction with subjective and objective conventional tools. Moreover, dialysis patients with nutritional risk or abnormal nutritional status assessed through traditional nutritional scales can be further evaluated for PEW by body composition analysis to confirm muscle mass status, ensuring that the anthropometric criterion for PEW is reliably fulfilled.

## 5 Limitations

This study has several limitations. First, its cross-sectional design precludes the establishment of causal relationships or temporal sequences between FFMI and clinical outcomes. Second, the sample size, though reasonable, is from a specific region in China, which may limit the generalizability of the findings. Larger multi-center prospective studies are needed to confirm our results. Third, while BIA is a practical and validated tool, the four-compartment model is considered the gold standard for body composition measurement but was not used here due to its complexity and cost. Furthermore, the proprietary equations in the BIA device, although widely used in research involving Chinese maintenance hemodialysis patients (15, 16), may not have been specifically validated for this population. We also reported hydration indicators (ultrafiltrate volume, TBW/FFM, ECW/TBW) and found only weak correlations with FFMI, but lacked a concurrent gold-standard method for hydration assessment (e.g., deuterium dilution) to definitively rule out its influence. Thus, the potential impact of hydration status on FFM estimates cannot be definitively excluded. Nonetheless, BIA remains a highly feasible and clinically relevant alternative.

## 6 Conclusion

In conclusion, low muscle mass is common but frequently underdiagnosed in MHD patients when assessed by conventional nutritional tools like ALB, BMI, NRS2002, and 7-point SGA. FFMI, as measured by BIA, provides an objective and potentially useful method and shows promise for improving the screening of PEW and sarcopenia in this vulnerable population. We recommend the adoption of regular BIA assessments as a supplementary objective measure to be used in conjunction with comprehensive nutritional evaluations in hemodialysis clinics to improve nutritional screening, facilitate timely nutritional intervention, and ultimately enhance patient prognosis.

## Data Availability

The raw data supporting the conclusions of this article will be made available by the authors, without undue reservation.

## References

[1] Gracia-Iguacel C Gonzalez-Parra E Barril-Cuadrado G Sanchez R Egido J Ortiz-Arduan A . Defining protein-energy wasting syndrome in chronic kidney disease: prevalence and clinical implications. Nefrologia. (2014) 34:507–19. doi: 10.3265/Nefrologia.pre2014.Apr.12522, 25036065

[2] Kopple JD. McCollum award lecture, 1996: protein-energy malnutrition in maintenance dialysis patients. Am J Clin Nutr. (1997) 65:1544–57. doi: 10.1093/ajcn/65.5.1544, 9129491

[3] Kalantar-Zadeh K Ikizler TA Block G Avram MM Kopple JD. Malnutrition-inflammation complex syndrome in dialysis patients: causes and consequences. Am J Kidney Dis. (2003) 42:864–81. doi: 10.1016/j.ajkd.2003.07.016, 14582032

[4] Kurajoh M Mori K Miyabe M Matsufuji S Ichii M Morioka T . Nutritional status association with sarcopenia in patients undergoing maintenance hemodialysis assessed by nutritional risk index. Front Nutr. (2022) 9:896427. doi: 10.3389/fnut.2022.896427, 35634393 PMC9137182

[5] Chan M Kelly J Batterham M Tapsell L. Malnutrition (subjective global assessment) scores and serum albumin levels, but not body mass index values, at initiation of dialysis are independent predictors of mortality: a 10-year clinical cohort study. J Ren Nutr. (2012) 22:547–57. doi: 10.1053/j.jrn.2011.11.002 22406122

[6] Leinig CE Moraes T Ribeiro S Riella MC Olandoski M Martins C . Predictive value of malnutrition markers for mortality in peritoneal dialysis patients. J Ren Nutr. (2011) 21:176–83. doi: 10.1053/j.jrn.2010.06.026, 21193323

[7] Ikizler TA Burrowes JD Byham-Gray LD Campbell KL Carrero JJ Chan W . KDOQI clinical practice guideline for nutrition in CKD: 2020 update. Am J Kidney Dis. (2020) 76:S1–S107. doi: 10.1053/j.ajkd.2020.05.006, 32829751

[8] Tan R Long J Fang S Mai H Lu W Liu Y . Nutritional risk screening in patients with chronic kidney disease. Asia Pac J Clin Nutr. (2016) 25:249–56. doi: 10.6133/apjcn.2016.25.2.24 27222407

[9] Cuppari L Meireles MS Ramos CI Kamimura MA. Subjective global assessment for the diagnosis of protein-energy wasting in nondialysis-dependent chronic kidney disease patients. J Ren Nutr. (2014) 24:385–9. doi: 10.1053/j.jrn.2014.05.004, 25106727

[10] Kyle UG Bosaeus I De Lorenzo AD Deurenberg P Elia M Gomez JM . Bioelectrical impedance analysis--part I: review of principles and methods. Clin Nutr. (2004) 23:1226–43. doi: 10.1016/j.clnu.2004.06.004 15380917

[11] Park JH Jo YI Lee JH. Clinical usefulness of bioimpedance analysis for assessing volume status in patients receiving maintenance dialysis. Korean J Intern Med. (2018) 33:660–9. doi: 10.3904/kjim.2018.197, 29961308 PMC6030410

[12] Carbone S Billingsley HE Rodriguez-Miguelez P Kirkman DL Garten R Franco RL . Lean mass abnormalities in heart failure: the role of sarcopenia, sarcopenic obesity, and Cachexia. Curr Probl Cardiol. (2020) 45:100417. doi: 10.1016/j.cpcardiol.2019.03.006, 31036371 PMC11146283

[13] Cederholm T Bosaeus I Barazzoni R Bauer J Van Gossum A Klek S . Diagnostic criteria for malnutrition - an ESPEN consensus statement. Clin Nutr. (2015) 34:335–40. doi: 10.1016/j.clnu.2015.03.001, 25799486

[14] Cederholm T Jensen GL Correia M Gonzalez MC Fukushima R Higashiguchi T . GLIM criteria for the diagnosis of malnutrition - a consensus report from the global clinical nutrition community. J Cachexia Sarcopenia Muscle. (2019) 10:207–17. doi: 10.1002/jcsm.12383, 30920778 PMC6438340

[15] Rong-Shao T Dan-Hua L Yan L Xiao-Shi Z Dong-Sheng Z Jing M. Bioelectrical impedance analysis-derived phase angle predicts protein-energy wasting in maintenance hemodialysis patients. J Ren Nutr. (2018) 29:295–301. doi: 10.1053/j.jrn.2018.09.001 30446269

[16] Shuai L Yuru Y Jingye S Limin M Yundan W Qin M . Total body water/fat-free mass ratio as a valuable predictive parameter for mortality in maintenance hemodialysis patients. Medicine (Baltimore). (2022) 101:e29904. doi: 10.1097/md.0000000000029904, 35945743 PMC9351861

[17] Johnson C Santos JA Sparks E Raj TS Mohan S Garg V . Sources of dietary salt in north and South India estimated from 24 hour dietary recall. Nutrients. (2019) 11:11. doi: 10.3390/nu11020318, 30717304 PMC6412427

[18] Landis JR Koch GG. The measurement of observer agreement for categorical data. Biometrics. (1977) 33:159–74. doi: 10.2307/2529310 843571

[19] Kang SS Chang JW Park Y. Nutritional status predicts 10-year mortality in patients with end-stage renal disease on hemodialysis. Nutrients. (2017) 9:9. doi: 10.3390/nu9040399, 28420212 PMC5409738

[20] Foucan L Merault H Velayoudom-Cephise FL Larifla L Alecu C Ducros J. Impact of protein energy wasting status on survival among Afro-Caribbean hemodialysis patients: a 3-year prospective study. Springerplus. (2015) 4:452. doi: 10.1186/s40064-015-1257-3, 26322258 PMC4549366

[21] Basic-Jukic N Radic J Klaric D Jakic M Vujicic B Gulin M . Croatian guidelines for screening, prevention and treatment of protein-energy wasting in chronic kidney disease patients. Lijec Vjesn. (2015) 137:1–8. 25906541

[22] Koefoed M Kromann CB Juliussen SR Hvidtfeldt D Ekelund B Frandsen NE . Nutritional status of maintenance dialysis patients: low lean body mass index and obesity are common, protein-energy wasting is uncommon. PLoS One. (2016) 11:e0150012. doi: 10.1371/journal.pone.0150012, 26919440 PMC4771706

[23] Ribeiro HS Neri SGR Oliveira JS Bennett PN Viana JL Lima RM. Association between sarcopenia and clinical outcomes in chronic kidney disease patients: a systematic review and meta-analysis. Clin Nutr. (2022) 41:1131–40. doi: 10.1016/j.clnu.2022.03.025, 35430544

[24] Marcelli D Usvyat LA Kotanko P Bayh I Canaud B Etter M . Body composition and survival in dialysis patients: results from an international cohort study. Clin J Am Soc Nephrol. (2015) 10:1192–200. doi: 10.2215/CJN.08550814, 25901091 PMC4491292

[25] Hwang SD Lee JH Lee SW Kim JK Kim MJ Song JH. Risk of overhydration and low lean tissue index as measured using a body composition monitor in patients on hemodialysis: a systemic review and meta-analysis. Ren Fail. (2018) 40:51–9. doi: 10.1080/0886022x.2017.1419963, 29347876 PMC6014525

[26] Jin M Du H Zhang Y Zhu H Xu K Yuan X . Characteristics and reference values of fat mass index and fat free mass index by bioelectrical impedance analysis in an adult population. Clin Nutr. (2019) 38:2325–32. doi: 10.1016/j.clnu.2018.10.010, 30389251

[27] Pan X Liu H Feng G Xiao J Wang M Liu H . Role of muscle mass and nutritional assessment tools in evaluating the nutritional status of patients with locally advanced nasopharyngeal carcinoma. Front Nutr. (2021) 8:567085. doi: 10.3389/fnut.2021.567085, 33763439 PMC7982395

[28] Luo Y Zhou L Li Y Guo S Li X Zheng J . Fat-free mass index for evaluating the nutritional status and disease severity in COPD. Respir Care. (2016) 61:680–8. doi: 10.4187/respcare.04358, 26814217

[29] Carrero JJ Chmielewski M Axelsson J Snaedal S Heimburger O Barany P . Muscle atrophy, inflammation and clinical outcome in incident and prevalent dialysis patients. Clin Nutr. (2008) 27:557–64. doi: 10.1016/j.clnu.2008.04.007, 18538898

[30] Kaysen GA Johansen KL Cheng SC Jin C Chertow GM. Trends and outcomes associated with serum albumin concentration among incident dialysis patients in the United States. J Ren Nutr. (2008) 18:323–31. doi: 10.1053/j.jrn.2008.04.002, 18558296 PMC3786208

[31] Alves FC Sun J Qureshi AR Dai L Snaedal S Barany P . The higher mortality associated with low serum albumin is dependent on systemic inflammation in end-stage kidney disease. PLoS One. (2018) 13:e0190410. doi: 10.1371/journal.pone.0190410, 29298330 PMC5752034

[32] Boesiger F Poggioli A Netzhammer C Bretscher C Kaegi-Braun N Tribolet P . Changes in serum albumin concentrations over 7 days in medical inpatients with and without nutritional support. A secondary post-hoc analysis of a randomized clinical trial. Eur J Clin Nutr. (2023) 77:989–97. doi: 10.1038/s41430-023-01303-w, 37419969 PMC10564620

[33] Honda H Qureshi AR Axelsson J Heimburger O Suliman ME Barany P . Obese sarcopenia in patients with end-stage renal disease is associated with inflammation and increased mortality. Am J Clin Nutr. (2007) 86:633–8. doi: 10.1093/ajcn/86.3.633, 17823427

[34] Scott D Sanders KM Aitken D Hayes A Ebeling PR Jones G. Sarcopenic obesity and dynapenic obesity: 5-year associations with falls risk in middle-aged and older adults. Obesity (Silver Spring). (2014) 22:1568–74. doi: 10.1002/oby.20734, 24585708

